# Misdiagnosis of scrotal and retroperitoneal lymphangioma in children

**DOI:** 10.1186/s12887-023-04380-9

**Published:** 2023-11-03

**Authors:** Wei Wu, Jiayu Mo, Kezhe Tan, Xingzhao Chen, WeiJue Xu, JiangBin Liu, Zhibao Lv

**Affiliations:** Department of General Surgery, Shanghai Children’s Hospital, School of Medicine, Shanghai Jiao Tong University, Shanghai, 200040 P. R. China

**Keywords:** Lymphangioma, Misdiagnosis, Inguinal area

## Abstract

**Background:**

Scrotal and retroperitoneal lymphangioma (SRL) in children is relatively rare and its clinical symptoms are usually difficult to distinguish from other conditions such as hydrocele and incarcerated inguinal oblique hernia. This study aimed to explore the clinical diagnosis and treatment of abdominal scrotal lymphangioma in children, and thus, to increase our understandings of this disease in clinical practice.

**Method:**

This study enrolled nine boys, aged 1–10, who were admitted to Shanghai Children’s Hospital from January 2019 to December 2020 and who were finally confirmed with lymphangioma in the inguinal area. The clinical manifestations, diagnosis, and treatment of these children were analyzed retrospectively. The length of diagnostic process ranged from 3 weeks to 20 months. We also reviewed other cases of initially misdiagnosed cases of SRL in English publications from 2000 to 2022.

**Results:**

The nine cases were misdiagnosed as hydrocele, hematoma, or inguinal hernia. Three patients received intracystic injection of bleomycin, three underwent laparoscopic mass resection, and three underwent resection of the inguinal lymphangioma under direct vision. Postoperative pathological analysis of the surgical specimens confirmed the diagnosis of benign cystic lesions and lymphangioma. Meanwhile, among the 14 cases of SRL in literature review, eight were misdiagnosed. Six were initially diagnosed as hydrocele, one as inguinal oblique hernia, and one as testicular tumor, all of which underwent ultrasonography scans. All cases were confirmed as lymphangioma after pathological examination.

**Conclusion:**

The non-specific clinical manifestations may contribute to the misdiagnosis of scrotal masses in children. A detailed and accurate medical history, careful physical examination, and imaging findings are important factors contributing to the preoperative differential diagnosis of scrotal lumps in children, but the final diagnosis is based on pathological examination.

## Background

Lymphangiomas are benign tumors originating from the mesoderm. Their formation is characterized by abnormal development of the lymphatic system and they may occur in the head and neck (75%), axilla (20%), retroperitoneum, scrotum, and inguinal area (5%) [1]. Although this tumor is generally benign, it tends to recur after treatment.

SRL is an extremely rare cause of scrotal lumps, which adds difficulties for practitioners to distinguish between SRL and other causes of scrotal masses, such as hydrocele, incarcerated inguinal hernia, and inguinal hematoma. Over the past 2 years, nine patients have sought medical advice in our center because of masses in the inguinal area. Of these, seven were initially misdiagnosed with hydrocele, one with incarcerated inguinal hernia, and one with inguinal hematoma, but all were proved to have lymphangioma by pathological staining after surgery.

This retrospective study aimed to summarize the clinical manifestations and pathological results of these nine patients, and to analyze the reasons for their initial misdiagnoses.

## Materials and methods

This study was approved by the Institutional Review Board of Shanghai Children’s Hospital, Shanghai Jiao Tong University, in accordance with the principles of the Declaration of Helsinki.

### General information

This research reviewed the cases of nine boys aged 1–10 years who presented at Shanghai Children’s Hospital with unilateral inguinal masses and who were eventually diagnosed with SRL from January 2019 to December 2020. The time from onset to final diagnosis ranges from 3 weeks to 20 months. Among these nine cases, seven were initially misdiagnosed as hydrocele, one as inguinal hematoma with a clear history of trauma, and one as incarcerated inguinal oblique hernia (Table 1). A diagnosis of lymphangioma was eventually confirmed in all nine cases by surgery.

**Table 1:** Details of nine patients with misdiagnosed abdominal scrotal lymphangiomas from January 2019 to December 2020

	Diagnosis	Age	Clinical manifestations	Mass size(cm)	transillumination experiment	Preoperative ultrasound	Treatment	Postoperative pathological tests
1	Hydrocele on the right,dysplasia of the left testis	12 M	Painless mass on the right scrotum	4 × 3 × 4	positive	Cystic lesions from right lower abdomen to inguinal area	Laparoscopic mass resection	Benign cystic disease, lymphangioma
2	Hydrocele on the left	1Y	Reversible mass in the inguinal area	4 × 3 × 2	positive	Cystic structure of left inguinal and left lower abdominal area	Laparoscopically assisted mass removal through external inguinal incision	Benign cystic disease, lymphangioma
3	Hydrocele on the right	3y	Painless mass in the right inguinal area	4 × 3 × 2	positive	Cystic structure of right inguinal	Resection of inguinal lymphangioma under direct vision	Benign cystic disease, lymphangioma
4	Hydrocele on the left	3y	Painless mass in the left inguinal area	4*3*2	positive	Cystic structure of left inguinal	Laparoscopic mass resection	Benign cystic disease, lymphangioma
5	Hydrocele on the right	4y	Painless mass in the left inguinal area	3 × 1 × 2	positive	Cystic lesions from left lower abdomen to inguinal area	Laparoscopy + bleomycin intratumoral injection treatment	Benign cystic disease, lymphangioma
6	Hydrocele on the left	5y	Reversible mass in the inguinal area	5 × 3 × 2	positive	Left communicating spermatic cord hydrocele	Resection of inguinal lymphangioma under direct vision	Benign cystic disease, lymphangioma
7	Hydrocele on the left	10Y	Painless mass in the left inguinal area	3 × 2 × 2	positive	Left communicating spermatic cord hydrocele	Laparoscopic exploration + bleomycin intrasaccular injection therapy	Benign cystic disease, lymphangioma
8	Left incarcerated inguinal hernia (Fig. 1)	17 M	Irreversible mass in the left inguinal area with pain and vomiting	4 × 5 × 4	Not performed	Left spermatic cord hydrocele	Laparoscopy + bleomycin intratumoral injection treatment	None
9	Hematoma in the left inguinal area	6Y	Mass in the left inguinal area with pain	3 × 4 × 4	positive	Mixed intensity mass in the left inguinal, inflammatory hematoma?	Resection of inguinal lymphangioma under direct vision	Lymphangioma

### Clinical evaluation

We recorded and analyzed the perioperative clinical manifestations, laboratory and imaging tests, and overall administration for these nine pediatric patients. Preoperative examinations were carried out, including routine blood, urine, and fecal tests, liver and kidney function, electrolytes, hepatitis B and C, and screening for sexually transmitted diseases. Ultrasound examination of the scrotum and inguen was also performed before surgery. Prognostic data were obtained by telephone follow-up.

## Results

Details of the nine misdiagnosed patients are presented in Table 1. Cases 1–7 were initially diagnosed at the first visit with hydrocele, Case 8 with inguinal hernia, and Case 9 with hematoma.

### Clinical manifestations

Three children (Cases 1, 3, and 7) who were misdiagnosed with hydrocele presented with a painless mass in the scrotum and inguinal area, which grew slowly and proved to be cystic, without tenderness on physical examination. The masses measured 4 × 3 × 4 cm, 4 × 3 × 2 cm, and 3 × 2 × 2 cm, respectively. Transillumination was positive and the masses could be squeezed and narrowed.

**Fig. 1:**
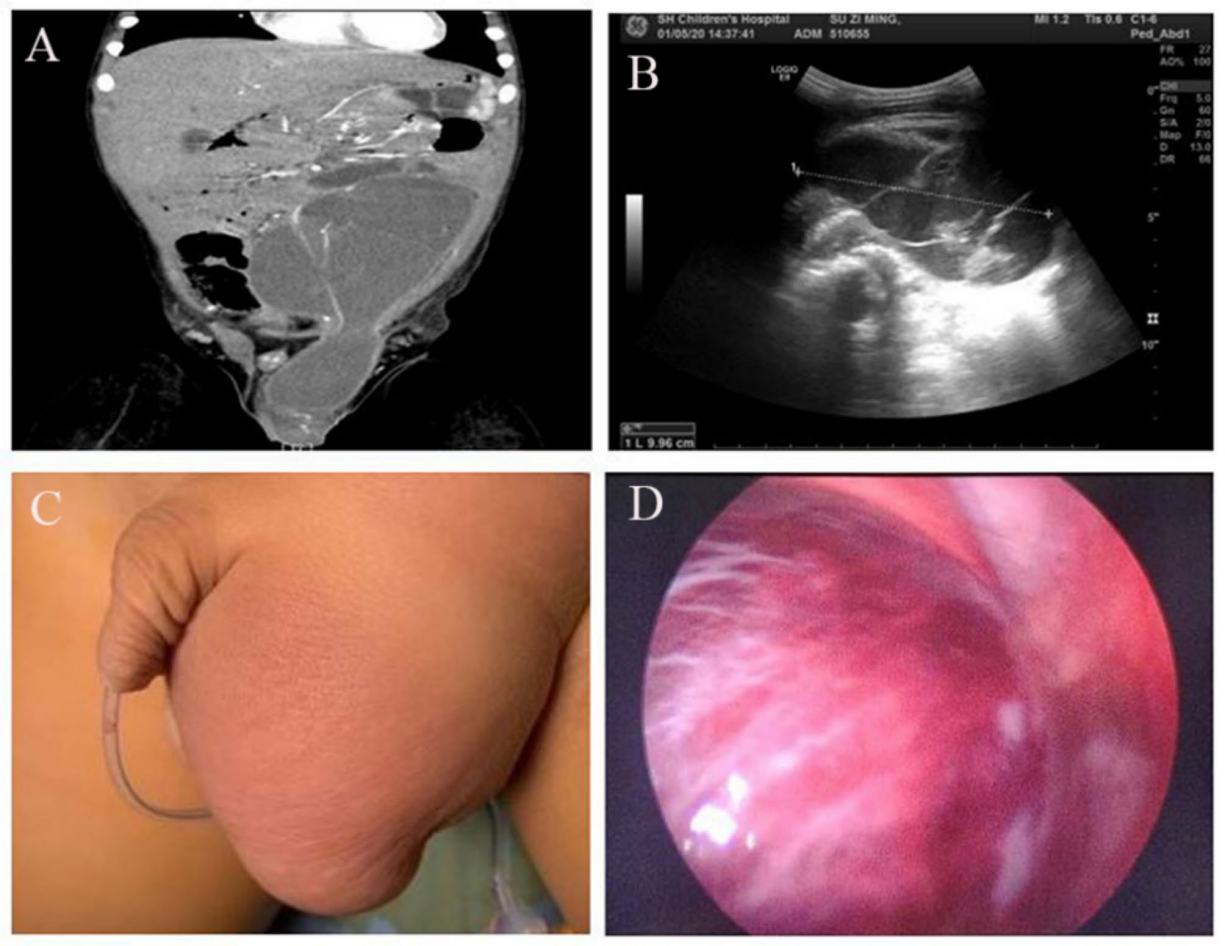
Patient (Case 8) misdiagnosed with an incarcerated inguinal hernia. **A**: Contrast-enhanced computed tomography scan; **B**: ultrasound image. **C**: The patient had a red, swollen, and painful mass measuring 4 × 5 × 4 cm in the left inguinal region before surgery. **D**: Surgical image

One child (Case 8) who was misdiagnosed with an incarcerated inguinal hernia displayed a red, swollen, and painful mass measuring 4 × 5 × 4 cm in the left inguinal region before surgery, having a hard texture and tenderness. The testis was slightly enlarged. However, the child vomited and refused to be touched and refused to cooperate with the transillumination examination (Fig. 1).

**Fig. 2:**
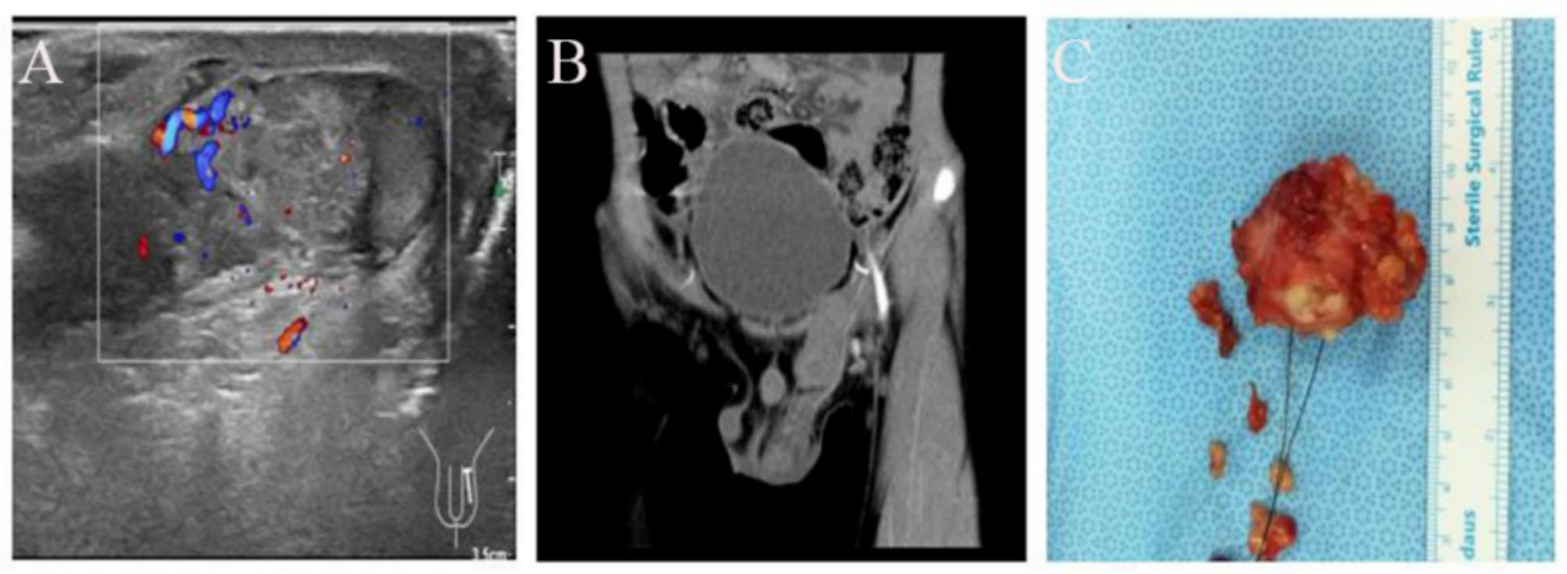
Patient (Case 9) misdiagnosed with a left inguinal hematoma. **A**: Ultrasound examination showed a mass with blood signals. **B**: Contrast-enhanced computed tomography scan. **C**: Surgically removed mass

One child (Case 9) who was misdiagnosed with a left inguinal hematoma had a clear history of trauma in the left inguinal area 1 week before surgery. He complained of pain in the left inguinal area, and we detected a tender 3 × 4 × 4 cm soft mass without redness. The transillumination results were positive (Fig. 2).

The other four cases misdiagnosed with hydrocele (Cases 2, 4, 5, and 6) were diagnosed as lymphangioma in the inguinal area before surgery.

### Laboratory and imaging tests

Cases 1, 3, and 7 underwent ultrasound examination of the inguinal area within 3 months before surgery, which suggested hydrocele.

One patient (Case 8) who was misdiagnosed with an incarcerated inguinal hernia appeared to show an inflammatory status and their brief complete blood count results were: white blood cells, 17.08 × 10^9^/L; hemoglobin, 88 g/L; and neutrophil percentage, 57.6%.

**Fig. 3:**
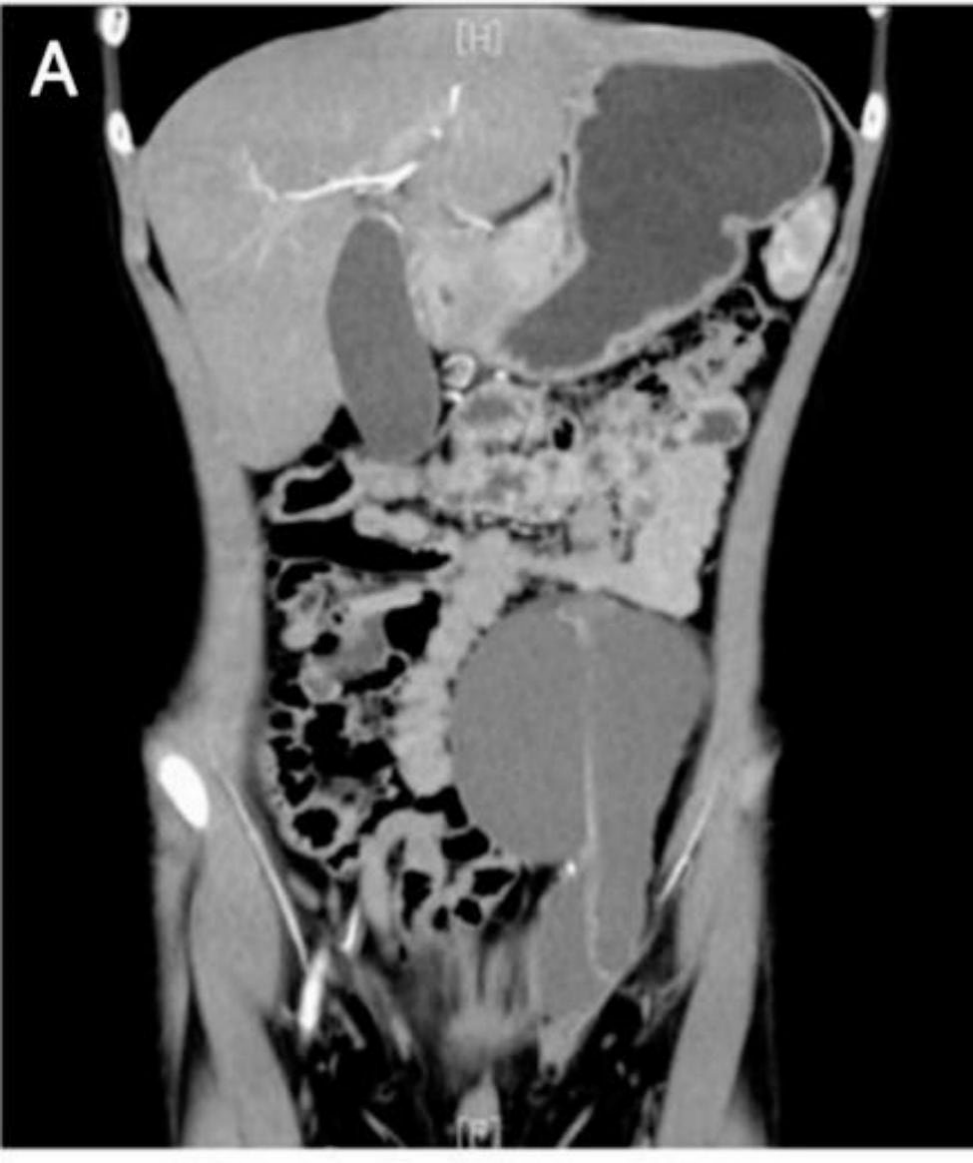
Contrast-enhanced computed tomography scan in Case 9 suggested the possibility of lymphangioma

Preoperative ultrasound imaging of Case 9 who was misdiagnosed with a left inguinal hematoma indicated a mixed mass in the left inguinal area, with the possibility of an inflammatory hematoma. The child also underwent preoperative contrast-enhanced computed tomography (CT), which showed mixed-intensity occupation, effusion, and bleeding (Fig. 3).

The remaining four cases (Cases 2, 4, 5, and 6) underwent ultrasound and pelvic CT and one (Case 4) also underwent magnetic resonance imaging, all of which suggested the possibility of lymphangioma.

### Treatment processes

Seven children (Cases 1, 2, 4, 5, 7, and 8) underwent exploratory laparotomy. Three patients with huge lymphangiomas originating from the retroperitoneum received intrasaccular injection of bleomycin with no allergic adverse reactions, and the other four underwent laparoscopic mass resection. Case 1 underwent laparoscopic radical resection of the mass after 2 months. Case 7, who had undergone left laparoscopic high ligation of the processus vaginalis 2 years previously, showed severely adhered tissue around the cyst during laparoscopic exploration 2 months later. In addition, the left spermatic cord blood vessel surrounded the cyst and the posterior wall adhered to the left iliac vessels. Considering the risks associated with cyst resection, bleomycin was smeared onto the cyst in this patient. In Case 2, a large, dark red tumor with high tension on the left retroperitoneum extended into the left scrotum during surgery and dark red, non-coagulated blood was found by puncture. In Case 4, laparoscopic separation was difficult and a transverse incision was made in the right scrotum, allowing complete removal of the mass after separation. In Case 5, the intra-abdominal cyst was separated and removed via a dermatoglyphic incision in the left inguinal area by laparoscope-assisted surgery. The vas deferens adhered tightly to parts of the cyst walls and bleomycin was therefore applied. A follow-up ultrasound examination of the inguinal area 1 month later showed no signs of cystic lymphangioma expansion (Table 1). This patient was suspected of having lymphangioma with bleeding, and bleomycin injection into the tumor was therefore performed.

Three patients (Cases 3, 6, and 9) underwent lymphangioma resection in the inguinal area under direct vision. In Case 9, the procedure was switched to inspection of the inguinal area under direct vision during surgery, which revealed an irregular bruising cyst closely linked to the spermatic cord and cremaster muscle. Considering a diagnosis of lymphangioma with hemorrhage, the tumor was resected completely.

Postoperative pathological tests of the surgical specimens showed benign cystic lesions attributed to lymphangioma in all patients.

The six children who underwent mass resection all had favorable prognoses at follow-up. In the three cases who received intrasaccular injection of bleomycin, the sizes of the tumors were obviously reduced and the patients were examined regularly by ultrasound every 2 months.

### Review of the literature

We also identified 14 cases of SRLs reported in the English literature from 2000 to 2022 (Table 2). Among these, eight cases were misdiagnosed preoperatively, including six as hydrocele, one as inguinal oblique hernia, and one as testicular tumor, all of whom were confirmed as lymphangioma by postoperative pathology. Most cases presented as painless masses in the scrotal and inguinal area, which could enlarge rapidly after trauma and were mostly associated with lymphangioma hemorrhage. One case presented with ascites and another with edema of the lower extremities. Transillumination results were not reported in most cases. All patients underwent preoperative ultrasonography, which suggested cystic septal lesions. Thirteen patients underwent surgical resection under direct vision, and one received OK-432 sclerotherapy because the lesion could not be completely removed. None of the children relapsed during the follow-up periods [2–13].

**Table 2:** Previously reported cases of SRL published in the English literature from 2000 to 2022

Author, year, reference	Diagnosis	Age	Clinical manifestations	Transillumination experiment	Ultrasound	Treatment	Postoperative pathological tests
Patoulias I, 2014 [2],	Inguinal hernia on the right	3.5 year-old	A painless soft mass in the right inguinal region	/	A cystic lesion injn the right. Hemi-scrotum	Surgery through a right inguinal incision	Cystic lymphangioma
Patoulias I, 2014 [2],	Hydrocele on the right	13-year-old	A painless mass at the right hemi-scrotum	/	A multi-cystic lesion attatched to the right epididymis	Surgery through scrotal approach	Cystic lymphangioma
Patoulias I, 2014 [2],	Lymphangioma	9-year-old	A 65 mm long mass in the left inguinal region(stable size until the age of 7 and then progressively enlarged)	/	A cystic lesion with 3 isolated compartments	Surgery through inguinal incision	Cystic lymphangioma
Ankit Parakh, 2009 [3]	Lymphangioma	4-year-old	A tense massive ascites and large hydrocele	/	Massive ascites	Resection of the mass through inguinal incision	Cystic Lymphangioma
Talal, 2009 [4],	Lymphangioma	3-year-old	A 4-month history of a gradullay enlarging,painless swelling in his left scrotum	Negative	A multilocular cystic structure	Resection of the mass through inguinal incision	Cystic Lymphangioma
Abdullahi Yusuf Ali, 2022 [5],	Hydrocele on the right	6-year-old	A painless,progressive enlargement of the right scrotum	Negative	Multiple cystic lesions in the right hemiscrotum extending to the proximal inguinal canal	Resection of the mass through the media raphe incision	Cystic lymphangioma
Hegde, 2018 [6]	Hydrocele on the right	11-month-old	A painless right scrotal swelling	Positive	A cystic mass 10*8 cm with multiple septae and lobulations	Resection of inguinal lymphangioma under direct vision	Cystic lymphangioma
Thayer, 2020 [7]	Hydrocele on the right	3-year-old	A painless swelling in the right hemiscrotum	Positive	Congential hydrocele	Resection of the mass through right transvers lower skin crease incision	Cystic lymphangioma
Vijay Kumar Kundal, 2012 [8]	Hydrocele on the right	8-year-old	A painless right scrotal swelling	Positive	A cystic mass with multiple septae of varying size	Resection of inguinal lymphangioma under direct vision	Lymphangioma
KEIICHI UCHIDA,2002 [9]	Lymphangioma	Neonate	A painless right flank and scrotal mass	Positive	A complex cystic mass in the right flank, retroperitoneal space, and right scrotum	OK-432 sclerotherapy	/
Häcker,2006 [10]	Lymphangioma	5-year-old	Acute scrotal pain and swelling	/	A cystic mass with multiple septae	Resection of inguinal lymphangioma under direct vision	Lymphangioma
Gulf J Oncolog. 2020 [11]	Hydrocele	5-year-old	A rapid increase in size within 1 week following the hydrocele surgery	/	Communicating hydrocele	Surgery through inguinal approach	Lymphangioma
Jelica Vikicevic, 2007 [12]	Lymphangioma	10-year-old	A reddish, lobulated scrotal swelling, left thigh edema	/	A multilobular, septated, cystic mass	Resection of inguinal lymphangioma under direct vision	Lymphangioma
Eric R. Weidman, 2002 [13]	Scrotal neoplasm	15-year-old	A painless left scrotal mass	/	A 1.5 × 2.5-cm multiseptated, cystic supratesticular mass	Resection of inguinal lymphangioma under direct vision	Lymphangioma

## Discussion

The patient’s medical history was not detailed. Lymphangiomas that present with bleeding and swelling may easily be confused with incarcerated inguinal oblique hernia. However, a detailed medical history may reveal a history of oblique hernia in children with an incarcerated inguinal oblique hernia, while children with a bleeding lymphangioma may have a history of trauma. Doctors should also be alerted to the possibility of lymphangioma in patients with recurrence after an inguinal oblique hernia or hydrocele.

Inadequate physical examination. In Case 2, despite the detection after exercise of a soft cystic mass that disappeared completely on squeezing, physical examination of the abdomen was not carried out and the mass was therefore misdiagnosed as a hydrocele. Abdominal scrotal lymphangiomas do not usually adhere to the skin and are soft and fluctuating with clear boundaries, showing positive results. However, transillumination could not be carried out in Case 3. In addition, infected or bleeding lymphangiomas may produce negative transillumination results. Some details of examinations are also helpful but may be easily overlooked, e.g., if the tension of the mass decreases after repeated compression, or stretching of the testicle or spermatic cord to observe significant changes in the mass may suggest that the mass is a hydrocele. Careful physical examination can thus help to avoid misdiagnosis.

Limitations of ultrasound examination. Most patients underwent ultrasound examination within 3 months before surgery, but the results were not sufficiently accurate. If the lymphangioma was limited to the scrotum and inguinal area without protruding into the abdominal cavity (e.g., Case 1), ultrasound suggested a cystic mass and misdiagnosed it as a hydrocele. In addition, if ultrasound fails to detect if the mass extends into the abdominal cavity at the inner ring orifice, or if abdominal ultrasound is not performed, it can also lead to misdiagnosis of lymphangiomas. In some cases, such as Case 3 and in some emergency cases, preoperative ultrasound cannot be completed to determine the presence of incarceration of the intestines or other features such as time constraints, leading to a misdiagnosis of incarcerated inguinal hernia.

Lymphangioma originates from the mesoderm and represents a congenital malformation of vascular development. It is not a ‘real’ tumor, but rather a benign lesion composed of multiple expanded lymphatic cysts formed by the abnormal development of the lymphatic system during the embryonic period. Lymphangiomas are more common in children, with about 90% diagnosed before the age of 2 years old. The incidences are equal between males and females [14, 15]. The pathophysiology of lymphangioma was first proposed by Whimster in 1976, who divided it into three types: capillary lymphangioma, cavernous lymphangioma, and cystic lymphangioma [16]. Cystic lymphangiomas mostly occur in the head, neck, and armpits (95%) and are soft, nonpulsatile, painless lumps. The surface skin is usually normal, but can have a bluish tint or pink vesicles resembling capillary malformations in some patients [17]. Lymphangiomas in the retroperitoneum, scrotum, and inguinal areas are rare. Singh et al. reported 32 cases of cystic lymphangioma in children, among which only one was in the scrotum [17]. In the early embryonic stage, the peritoneum of the lower abdomen in males forms a protrusion (processus vaginalis) towards the inguinal region and extends along the inguinal canal to the bottom of the scrotum. If the processus vaginalis is not closed, a lymphangioma in the abdominal cavity can reach the scrotum through the inguinal canal, and is thus often misdiagnosed as an indirect inguinal hernia, hydrocele, testicle torsion, or varicocele [18].

Painless scrotal masses are usually benign, but a few are malignant, such as sarcomas [13]. Scrotal lymphangiomas are uncommon benign masses of the scrotum caused by inadequate drainage of isolated lymphatic vessels, often presenting as a painless mass that grows gradually over time [3, 19, 20]. Masses in the subcutaneous tissue of the scrotum are not linked to the spermatic cord and may show invasive growth, low tension, and unclear borders and are thus often confused with hernia, hydrocele, and varicocele. If the cyst hemorrhages (spontaneously or after trauma), inflammation occurs, or the balance between lymphatic production and drainage is disrupted, the initially painless mass can suddenly and rapidly enlarge, leading to the acute onset of pain, and potential misdiagnosis of acute testicle torsion [6, 11, 12]. Abdominal scrotal lymphangiomas in children are mostly cystic soft tissue masses. Ultrasound is superior to CT for observing the internal structure of the lesion, which shows subcutaneous mesh occupying behind the testis. Color Doppler flow imaging can also indicate low-velocity venous blood-flow signals on the cyst wall of the scrotal lymphangioma and the inner compartment. If the cyst bleeds, light spots may be seen floating in the dark area [21, 22]. If the doctor notes other symptoms besides scrotal swelling, such as skin abnormalities, a large lump that does not change with body posture and a lobular appearance, ultrasound examination should be performed. If ultrasound examination reveals multilocular cystic masses in the inguinal area or the scrotum, the abdominal and pelvic cavity should be routinely scanned and imaging examinations such as CT or magnetic resonance imaging are also recommended. Puncture can also be used to aid diagnosis: the puncture fluid from lymphangiomas growing in deep soft tissue is light yellow, but this becomes dark red and non-coagulated when combined with internal bleeding [23]. The gold standard for diagnosis is pathological examination. Lymphangiomas are often covered with squamous epithelium, smooth muscle fibers, blood vessels, fat, and lymphoid tissues, and immunohistochemical staining for D2-40 is mostly positive.

Various treatments are available for abdominal scrotal lymphangiomas. Surgical treatment with an inguinal approach is usually the first consideration [24–26]. During surgery, part of the cyst fluid is aspirated to reduce the tension of the lymphangioma, and the lymphangioma plus the involved skin and tissues are then removed as completely as possible, to prevent recurrence. However, polycystic lymphangiomas are difficult to separate from the surrounding tissues and have a higher risk of bleeding and secondary damage due to the involvement of multiple tissue planes, large size, and high tension. Resection in these cases is usually incomplete, with recurrence rates as high as 35–64%, which could drop to 17–22% with complete resection [27]. Some researchers believe that lymphangiomas in infants may regress spontaneously and small asymptomatic lesions may thus be treated conservatively, while symptomatic lesions need to be managed actively, but preferably using less-traumatic options than surgery [15, 28]. Sclerotherapy, i.e., drug-injection therapy, has become a mainstream option, with a high success rate and low rates of recurrence and complications [29]. If the cyst cannot be completely resected during surgery, it will continue to produce lymphatic fluid, leading to recurrence. Sclerotherapy can thus act on the endothelial cells of blood vessels and lymphatic vessels resulting in luminal embolization and fibrosis, inhibiting lymphatic fluid secretion and reducing the recurrence rate [30]. Uchida et al. noted that lymphangiomas in the inguinal scrotum often infiltrated the surrounding tissues and main structures, such as the vas deferens, making radical surgical resection difficult and increasing the recurrence and morbidity rates. They reported the case of a huge retroperitoneal lymphangioma extending into the scrotum through the inguinal canal, which was successfully treated with OK-432 sclerotherapy, leading to a good outcome [9]. OK-432 is a preparation comprising heat-treated and freeze-dried group A hemolytic Streptococcus penicillin. It has shown good efficacy for lymphangioma [31], but its main adverse reaction is transient fever after injection, with almost 100% of patients experiencing fever within 6 h after injection, lasting for 2–4 days. Most patients also experience serious local reactions such as erythema and swelling of the affected area, while a few may have symptoms of shock. Multiple treatments are therefore required for OK-432 to achieve satisfactory therapeutic effects [32]. Sclerosants such as bleomycin and peplomycin are also widely used. These are cytotoxic antibiotics extracted from Streptococcus, which have demonstrated good effects at low doses; however, complications include interstitial pneumonia and pulmonary fibrosis, with potentially serious consequences [33]. This type of drug is also commonly used in our institution. Three patients in the current study were treated with bleomycin applied to the cysts after surgery, with no occurrence of pulmonary fibrosis during follow-up. Non-selective beta-blockers, such as propranolol, are also commonly used for treating hemangiomas, with proven efficacy for these types of vascular malformations. Regular monitoring of the patient’s heart rate, blood glucose, and blood pressure is required during the treatment process. However, the effectiveness of these drugs for lymphangiomas is not clear, and they are not currently used for the clinical treatment of lymphangioma [34].

Pediatric abdominal scrotal lymphangioma is relatively rare and its clinical symptoms are often difficult to distinguish from those of other conditions such as hydrocele and incarcerated inguinal hernia. The diagnosis therefore depends mainly on a detailed and accurate medical history, careful physical examination, and imaging and pathological tests. Information on peritoneal scrotal lymphangioma is currently lacking. Unilocular peritoneal scrotal lymphangiomas can be resected directly and completely under laparoscopy, while surgical resection of multilocular lymphangiomas may damage the surrounding tissues, such as the vas deferens. In these case, sclerotherapy may be a safe and effective method, resulting in a favorable prognosis.

This study was limited by the small number of cases, which meant that we were unable to verify the conclusions. In the future, we aim to compare the generalized patterns with clinical cases and further validate their practical value.

## Conclusions

SRL in children is a rare disease and its non-specific clinical manifestations lead to a high risk of its misdiagnosis among patients with scrotum masses as non-SRL conditions, including hydrocele and incarcerated inguinal oblique hernia. It is therefore necessary to obtain a detailed and accurate medical history and carry out careful physical examination, imaging, surgery, and pathological tests to make an accurate diagnosis. Unilocular peritoneal scrotal lymphangioma can be resected directly and completely under laparoscopy, while sclerotherapy is a safe and effective method for patients with multilocular lymphangioma.

## Data Availability

The datasets used and/or analyzed during the current study are available from the corresponding author on reasonable request.

## List of abbreviations

CT: Computed tomography
SRL: Scrotal and retroperitoneal lymphangioma
